# Behavioural divergence of sympatric *Anopheles funestus* populations in Burkina Faso

**DOI:** 10.1186/1475-2875-13-65

**Published:** 2014-02-24

**Authors:** Wamdaogo M Guelbeogo, N’Fale Sagnon, Fang Liu, Nora J Besansky, Carlo Costantini

**Affiliations:** 1 Centre National de Recherche et de Formation sur le Paludisme, Ouagadougou 01 BP 2208, Burkina Faso; 2 Department of Applied and Computational Mathematics and Statistics, University of Notre Dame, Notre Dame, IN 46556, USA; 3 Eck Institute for Global Health, Department of Biological Sciences, University of Notre Dame, Notre Dame, IN 46556, USA; 4 Institut de Recherche pour le Développement (IRD), Unité Mixte de Recherche MIVEGEC (UM1, UM2, CNRS 5290, IRD 224), Centre IRD de Montpellier 956, Avenue Agropolis BP 64501, Montpellier Cedex 5 34394, France

**Keywords:** *Anopheles funestus*, Anthropophily, Behavioural divergence, Chromosomal forms, Exophily, Folonzo, Kiribina, Malaria vector, West Africa

## Abstract

**Background:**

In Burkina Faso, two chromosomal forms of the malaria vector *Anopheles funestus*, Folonzo and Kiribina, are distinguished by contrasting frequencies of shared polymorphic chromosomal inversions. Sympatric and synchronous populations of Folonzo and Kiribina mate assortatively, as indicated by a significant deficit of heterokaryotypes, and genetic associations among inversions on independently segregating chromosome arms. The present study aimed to assess, by intensive longitudinal sampling, whether sympatric Folonzo and Kiribina populations are characterized by behavioural differences in key malaria vectorial parameters.

**Methods:**

The study was conducted in two adjacent villages near Ouagadougou, in the dry savanna of central Burkina Faso. Mosquito adult resting behaviour of both forms was compared based on parallel indoor/outdoor collections across six breeding seasons; 8,235 fully karyotyped samples of half-gravid females were analysed in total. Additionally, indoor/outdoor human biting behaviour, host selection, and *Plasmodium falciparum* sporozoite rate was assessed and compared between chromosomal forms.

**Results:**

The Kiribina form was numerically predominant in the area. However, the Folonzo form was significantly over-represented in indoor resting collections and showed stronger post-prandial endophily, while Kiribina predominated outdoors. Neither form was statistically distinguishable in human biting behaviour, and both were more likely to seek human blood meals indoors than outside. The human blood index and sporozoite rate were comparably high in both chromosomal forms in indoor collections (>89% and >8%, respectively).

**Conclusions:**

Both Kiribina and Folonzo chromosomal forms are formidable malaria vectors in Burkina Faso. However, the significantly greater tendency for the Kiribina form to rest outdoors despite its pronounced anthropophily suggests that uniform exposure of the overall *An. funestus* population to indoor-based vector control tools cannot be expected; Kiribina is more likely to evade indoor interventions and escape unharmed outdoors, reducing the efficacy of malaria control. Accordingly, more efficient methods to detect Kiribina and Folonzo, and a more complete understanding of their distribution and behaviour in Africa are advocated.

## Background

The malaria vectorial system in tropical Africa is dominated by four species of major importance, *Anopheles gambiae*, *Anopheles coluzzii*, *Anopheles arabiensis* and *Anopheles funestus,* which are broadly codistributed across much of tropical Africa in close association with humans [1,2]. The first three species belong to the same cryptic species complex (the *An. gambiae* complex) whose members cannot be distinguished morphologically at any developmental stage, although they differ in aquatic larval ecology and adult behaviours relevant to malaria transmission and control (e.g., degree of anthropophily and tendency to blood-feed or rest indoors) [3,4]. *Anopheles funestus* and its presently recognized closest relatives are classified into a group and subgroup [5,6] rather than a species complex, owing to slight morphological distinctions mainly at immature stages. However, further cryptic taxonomic complexities within the group have recently come to light and more can be anticipated as *An. funestus* research emerges from a period of neglect [7-11]. Malaria transmission by the Funestus Subgroup is overwhelmingly attributed to *An. funestus sensu stricto*, owing to its strong preference for human blood meals (see reviews by [7,12]).

*Anopheles funestus s.s.* is characterized by abundant genetic polymorphism, exemplified by at least 17 chromosomal re-arrangements segregating within and among populations across Africa [13,14]. Although this species is generally considered to be uniformly anthropophilic and endophilic throughout its range, complex and incompletely understood patterns of population structure based on cytogenetic and DNA markers have been detected [15-20]. In particular, two chromosomal forms designated “Folonzo” and “Kiribina” have been described in West Africa [16]. First discovered in Burkina Faso and most intensively characterized in that country, Folonzo and Kiribina populations carry markedly different frequencies of shared polymorphic chromosomal inversions, mainly involving arm 3R [16,17]. In localities where the chromosomal forms are synchronous and stably sympatric across successive breeding seasons and years, there are highly significant departures from Hardy Weinberg equilibrium and significant genetic associations among physically unlinked inversion systems; alternative homokaryotypes are more frequent than expected under random mating, and there are significant deficits of heterozygotes in virtually all population samples, consistent with assortative mating by form [16,17].

Neither inversions nor inversion combinations are diagnostic taxonomic characters. However, the Kiribina form is predominantly homokaryotypic for the standard chromosomal arrangements, while Folonzo, the more chromosomally polymorphic taxon, carries high frequencies of inversions 3Ra and 3Rb, and presumably corresponds to *An. funestus* from East Africa, where Kiribina has not been recorded [16]. Strongly reminiscent of the chromosomal forms of *An. gambiae*[21], these alternative karyotypes show cyclical patterns of seasonal variation in relative abundance linked to temperature and rainfall, likely reflecting differences in geographic distribution modulated by larval habitat utilization [22]. Although direct evidence is lacking, the Folonzo form is associated with natural larval habitats such as marshes, while Kiribina is associated with larval habitats created by the practice of agriculture, notably rice fields. Molecular genetic studies using mtDNA and microsatellite markers revealed very slight but significant divergence between sympatric samples of Folonzo and Kiribina across Burkina Faso, although nuclear divergence was not genome-wide and could be explained by loci on chromosome 3R inside and outside inversions [23,24]. These data are suggestive of an incipient process of ecological divergence and lineage splitting, similar to, but less advanced than, that responsible for the divergence of *An. coluzzii* and *An. gambiae* (formerly recognized as chromosomal or molecular forms [25,26]).

Previous studies in Burkina Faso and Senegal have reported similarly high rates of anthropophily and comparable *Plasmodium falciparum* infection rates in sympatric Folonzo and Kiribina populations [23,24,27]. However, there were indications of differences in indoor resting behaviour, leading to the suggestion that the Kiribina form may be more easily diverted to outdoor resting and biting, particularly in localities where alternative hosts such as cattle outnumber the local human population [16]. If the ecological and genetic heterogeneities between Folonzo and Kiribina indeed extend to behavioural differences of importance to malaria epidemiology and control, these vectorial differences must be understood more deeply. Toward that end, resting and biting behaviour were assessed separately for sympatric and synchronous Folonzo and Kiribina populations in the rural villages of Kuiti and Koubri near Ouagadougou, Burkina Faso. Observations spanned six breeding seasons and 8,235 fully karyotyped Folonzo and Kiribina adult half-gravid females.

## Methods

### Study area

The study was carried out in the arid Sudan savanna vegetation belt of West Africa, in adjacent rural villages located 35 km south of Ouagadougou, the capital of Burkina Faso. Koubri (12°11′54 N; 1°23′43 W) and Kuiti (12°11′36 N; 1°23′11 W) lie about 1 km apart on opposite margins of an artificial lake bordered by permanent swamps. A detailed map and additional information about the study area can be found elsewhere [17,24]. In this region, the *An. funestus* breeding season commences at the end of the rainy season (September), extends throughout the cool dry season (October-February), and ends in April, mid-way through the hot dry season (March-May). Folonzo peaks in relative abundance following the rains, in October-December [22].

### Chromosomal form identification

Adult Funestus Group females were sorted morphologically [28] in the field under a dissecting microscope. Ovaries from females at the appropriate gonotrophic stage were immediately dissected and preserved by individual mosquito in 1.5 ml microcentrifuge tubes using Carnoy’s fixative (ethanol:glacial acetic acid, 3:1), while the associated carcass was placed in a correspondingly labelled microcentrifuge tube with desiccant. Molecular taxonomic identification of *An. funestus* based on DNA extracted from the carcass was performed with a modified rDNA-based PCR assay [24]. Ovaries in Carnoy’s were held on ice until they could be stored at -20°C for later polytene chromosome analysis. Polytene chromosomes of *An. funestus* were spread [29] and examined under a phase-contrast microscope. Karyotypes were assigned using the cytogenetic map of Sharakhov *et al.*[13]. Of all karyotyped samples, 92% were successfully scored for all inversions. Chromosomal form assignment followed the deterministic algorithm of Guelbeogo *et al*. [17]. Using a probabilistic assignment test as an alternative method of classification of karyotypes sampled from the same localities as the present study, these authors estimated the rate of mis-classification to be very low, about 0.7%.

### Resting behaviour

An estimate of the odds of adult females of the Folonzo or Kiribina forms resting indoors was calculated by comparing the relative abundance of each form in resting collections that were conducted indoors and outdoors in parallel. Indoor resting mosquitoes were sampled in the afternoon inside multiple huts and compounds in both villages, by insecticide spray-sheet catches (ISC) three times per week; mosquitoes resting outdoors in the villages were sampled at least twice weekly from four Muirhead-Thompson style pit-shelters with manual aspirators [30]. In addition, an estimate of the odds of post-prandial indoor-resting by outdoor-biting Folonzo or Kiribina was calculated based on blood meal identifications performed on indoor/outdoor-resting collections made between 2005–2007 (described below). As cattle do not share the domestic environment with humans in the study area, mosquitoes with exclusively bovine blood meals must have fed outdoors on cattle. Accordingly, the numbers of each form that fed solely on cattle were compared between indoor-resting (ISC) and outdoor-resting (PIT) collections from the same time period.

### Human biting behaviour

Human biting behaviour of Folonzo and Kiribina was assessed by human landing catches (HLC). Two teams of trained collectors worked in two different compounds in eight-hour shifts (21:00–05:00), twice per week from October 2002-January 2003. Each team consisted of a pair of collectors, one of whom performed an indoor landing catch while the other did the same outdoors, reversing positions on a subsequent night to control for collector-specific effects. To identify the chromosomal form of host-seeking *An. funestus* captured by HLC, mosquitoes were blood-fed on rabbits the same night of capture, and held in the insectary until they reached the stage of ovarian development appropriate for polytene chromosome analysis.

### Blood meal identification and *Plasmodium falciparum* detection

Samples of blood-fed mosquitoes collected in the 2005–2006 and 2006–2007 breeding seasons were cut transversely between the thorax and the abdomen. The origin of the blood meal (human, bovine, mixed) in the posterior portion was identified by an enzyme-linked immunosorbent assay (ELISA) using specific monoclonal antibodies. The presence of *P. falciparum* circumsporozoite protein (CSP) in the anterior portion (head + thorax) also was detected by ELISA in these samples.

### Data analysis

The human blood index (HBI) of each chromosomal form was calculated as the proportion of human and mixed blood meals identified relative to all blood meals identified by ELISA in samples of that form. The sporozoite rate of each form was calculated as the proportion of mosquitoes in a sample that were positive for *P. falciparum* CSP by ELISA. The odds ratio (OR; [31]), the ratio of the odds of an event occurring in one group to the odds of it occurring in another, was used to compare vectorial parameters between the chromosomal forms. The precision of the OR was estimated using the 95% confidence interval (CI). *P*-values are reported based on the Pearson Chi-square test of association for 2×2 contingency tables, with *P* <0.05 considered as significant.

### Ethical approval

The study protocols were reviewed and approved by the institutional health ethical review board of Burkina Faso. Informed consent was obtained from participants.

## Results

Resting behaviour by sympatric populations of the two chromosomal forms of *An. funestus* was assessed by parallel indoor/outdoor collections across six breeding seasons in two adjacent rural villages located in the dry savanna of central Burkina Faso. The results from the 8,235 fully karyotyped samples are presented in Table 1. Samples of the Kiribina form were generally larger than corresponding samples of the Folonzo form both indoors and out, particularly for the outdoor collections. However, during most breeding seasons and for the pooled samples, the Folonzo form was more likely than Kiribina to rest inside human dwellings rather than outside in pit-shelters. For 50% of the seasons, particularly when the numbers of outdoor-resting mosquitoes were sufficiently large, a Chi-square test of association for a 2x2 contingency table indicated that the stronger indoor resting tendency of Folonzo relative to Kiribina was statistically significant (Table 1).

**Table 1:** **Resting behaviour of *Anopheles funestus *** **chromosomal forms in Burkina Faso**

**Season**	**Sample**	**Total**	**Folonzo**	**Kiribina**	**OR**	**0.95 CI**	***P***
1999-2000	ISC/indoor	1154	377	777	1.94	1.29-2.93	0.001
	PIT/outdoor	155	31	124			
2000-2001	ISC/indoor	1164	208	956	1.58	0.55-4.54	NS
	PIT/outdoor	33	4	29			
2001-2002	ISC/indoor	2733	659	2074	0.95	0.49-1.84	NS
	PIT/outdoor	48	12	36			
2002-2003	ISC/indoor	485	301	184	3.03	1.70-5.37	<0.0001
	PIT/outdoor	57	20	37			
2005-2006	ISC/indoor	99	63	36	2.33	0.49-11.02	NS
	PIT/outdoor	7	3	4			
2006-2007	ISC/indoor	932	343	589	11.87	8.93-15.76	<0.0001
	PIT/outdoor	1368	64	1304			
Pooled	ISC/indoor	6567	1951	4616	4.84	4.02-5.82	<0.0001
	PIT/outdoor	1668	134	1534			

OR, odds ratio of indoor/outdoor resting by Folonzo *versus* Kiribina; CI, confidence interval; ISC, insecticide spray-sheet catch; PIT, Muirhead-Thomson pit-shelter [30].

A measure of post-prandial resting behaviour by outdoor-feeding *An. funestus* was estimated by focusing on those mosquitoes with exclusively bovine-derived blood meals and comparing their numbers between indoor-resting and outdoor-resting samples of each chromosomal form. Based on this measure, the Folonzo form was significantly more likely than Kiribina to rest indoors following a bovine blood meal taken outdoors (Table 2).

**Table 2:** **Post-prandial resting behaviour of outdoor-feeding *Anopheles funestus *** **chromosomal forms in Burkina Faso (2005–2007)**

Sample*	**Total**	**Folonzo**	**Kiribina**	**OR**	**0.95 CI**	***P***
ISC/indoor	38	9	29	5.64	2.43-13.10	0.0003
PIT/outdoor	518	27	491			

OR, odds ratio of post-prandial indoor resting by Folonzo *versus* Kiribina for outdoor-feeding *An. funestus* (2005–2007 season); CI, confidence interval; PIT, Muirhead-Thomson pit-shelter [30]; ISC, insecticide spray-sheet catch.

*Includes only *An. funestus* having blood fed exclusively on cattle, based on blood meal identification.

HLC, conducted in parallel indoors and outdoors during the 2002–2003 breeding season, were used to compare human-biting behaviour between the chromosomal forms. Based on the >1,000 female mosquitoes captured, karyotyped and assigned to chromosomal form in 2002–3, human biting behaviour indoors *versus* outdoors was indistinguishable between chromosomal forms (Table 3). For both, the proportion of mosquitoes seeking human blood meals indoors *versus* outdoors was higher and of a similar magnitude. Importantly, the absolute numbers of the Folonzo form captured by HLC, both indoors and out, were larger than those from the corresponding Kiribina samples. These observations suggest that the Kiribina form may be more opportunistic, and the Folonzo form more anthropophilic, in host-seeking behaviour.

**Table 3:** **Human biting behaviour of *Anopheles funestus *** **chromosomal forms in Burkina Faso (2002–2003)**

**Month**	**Sample**	**Total**	**Folonzo**	**Kiribina**	**OR**	**0.95 CI**	***P***
October	HLC/indoor	363	263	100	0.87	0.54-1.40	NS
	HLC/outdoor	117	88	29			
November	HLC/indoor	218	151	67	1.18	0.74-1.87	NS
	HLC/outdoor	131	86	45			
December	HLC/indoor	70	42	28	0.62	0.30-1.30	NS
	HLC/outdoor	58	41	17			
January	HLC/indoor	80	45	35	1.63	0.73-3.65	NS
	HLC/outdoor	34	15	19			
Pooled	HLC/indoor	731	501	230	1.04	0.79-1.37	NS
	HLC/outdoor	340	230	110			

OR, odds ratio of indoor/outdoor human biting by Folonzo *versus* Kiribina; CI, confidence interval; HLC, human landing catch.

Host selection was assessed by blood meal identification during the 2005–2006 and 2006–2007 breeding seasons. The indoor resting samples of Folonzo and Kiribina both had a relatively high human blood index, 95.9 and 89.3%, respectively (Table 4). Folonzo was the form more likely to have fed on humans in whole or in part, rather than solely on cattle (*P* <0.006), but this trend may reflect differences between forms in post-prandial resting behaviour rather than differences in the intrinsic preference for human hosts (i.e., host choice). While the size of indoor samples was balanced between Folonzo and Kiribina, there was a large skew in outdoor resting sample size between the forms, only 30 for Folonzo compared to 529 for Kiribina, reflecting the greater tendency for the latter form to rest outdoors. Both outdoor resting samples had drastically lower human blood indices, 10 and 7% for Folonzo and Kiribina, respectively. Folonzo remained the form more likely to have fed on humans than cattle (OR, 1.44), although this trend was not statistically significant.

**Table 4:** **Host selection of *Anopheles funestus *** **chromosomal forms in Burkina Faso (2005–2007)**

**Sample**	**Blood meal**	**Folonzo**	**Kiribina**	**OR**	**0.95 CI**	***P***
ISC/indoors	Human + Mixed	191 + 21 (212)	232 + 11 (243)	2.81	1.30-6.07	0.006
	Bovine	9	29			
	Total	221	272			
	HBI	95.9%	89.3%			
PIT/outdoors	Human + Mixed	2 + 1 (3)	13 + 25 (38)	1.44	0.42-4.95	NS*
	Bovine	27	491			
	Total	30	529			
	HBI	10.0%	7.2%			

*NS by Fisher Exact test. Chi-square not calculated due to an expected cell frequency below 5.

OR, odds ratio of human/bovine blood meal by Folonzo *versus* Kiribina; CI, confidence interval; ISC, insecticide spray-sheet catch; PIT, Muirhead-Thomson pit-shelter [30]; Mixed, blood meal of human and bovine origin; HBI, human blood index.

During the same 2005–2007 seasons that host selection was evaluated, samples were analysed for *P. falciparum* infection by testing for the presence of the CSP in indoor and outdoor resting samples of the two chromosomal forms. Infection rates in the indoor resting samples did not differ significantly between forms, being similarly high in both (8.5-8.8%; Table 5). Among the outdoor resting mosquitoes, the small Folonzo sample contained no sporozoite-positives, while the much larger Kiribina sample contained 32 (3%) sporozoite positives.

**Table 5:** ***Plasmodium falciparum *** **sporozoite rate of *Anopheles funestus *** **chromosomal forms in Burkina Faso (2005–2007)**

**Sample**	**CSP**	**Folonzo**	**Kiribina**	**OR**	**0.95 CI**	***P***
ISC/indoor	+	27	45	0.97	0.59-1.59	NS
	-	291	469			
	Total	318	514			
	%CSP+	8.5%	8.8%			
PIT/outdoor	+	0	32	0.00	0.00-2.14	NS*
	-	61	1035			
	Total	61	1067			
	%CSP+	0.0%	3.0%			

*NS by Fisher Exact test.

CSP, circumsporozoite protein; OR, odds ratio of sporozoite infection in Folonzo *versus* Kiribina; CI, confidence interval; ISC, insecticide spray-sheet catch; PIT, Muirhead-Thomson pit-shelter [30].

## Discussion

Intensive longitudinal sampling of *An. funestus* from adjacent villages in the Sudan savanna of Burkina Faso, West Africa, affirms and extends the previous findings by Costantini *et al.*[16] of behavioural divergence between sympatric and synchronous chromosomal forms known as Folonzo and Kiribina. The high rate of anthropophagy by both forms (>89% of indoor samples), coupled with comparably high rates of *P. falciparum* infection (>8% of indoor samples) emphasize the fact that Folonzo and Kiribina both are formidable malaria vectors in this part of Africa. The Kiribina form often outnumbered Folonzo. Yet, Folonzo was disproportionately represented in indoor *versus* outdoor resting samples and was more inclined to post-prandial endophily, while Kiribina was over-represented outdoors in pit shelters. This suggests that the overall *An. funestus* population is not uniformly exposed to indoor-based malaria interventions such as insecticide-treated nets and house spraying by residual insecticides, and that those indoor interventions are less effective against the Kiribina form.

There is precedence for chromosomal inversion-associated heterogeneity in mosquito resting behaviour in the West African savanna, uncovered by Coluzzi and colleagues through polytene chromosome analysis of *An. gambiae* and *An. arabiensis* populations during the Garki Project in Nigeria [3,32]. Such behavioural heterogeneity was responsible for the failure to interrupt malaria transmission during the course of the Project, despite rigorous insecticide applications and simultaneous administration of anti-malarial drugs to the human population [33]. Indeed, there are hints that this same phenomenon has been witnessed previously with respect to *An. funestus* in the West African savanna, where Kiribina co-exists with Folonzo. In the absence of Kiribina in eastern and southern Africa, historical house spraying campaigns not only locally eliminated *An. funestus*, but the effect was maintained for several years following the cessation of spraying, due to the apparent inability of *An. funestus* to recolonize some areas [34]. Likewise, *An. funestus* was eliminated from humid forest and degraded forest areas in West Africa where malaria is meso- or hypo-endemic [34], an environment where Folonzo is predicted to dominate [16,35-37]. However, in the savannas of West Africa where malaria is holo- or hyperendemic, similar historical indoor spraying campaigns failed to eliminate the species [34]. Exophilic populations persisted which, despite marked anthropophily, continued to feed outdoors on cattle as well as humans, and also entered sprayed houses to bite humans, but escaped unharmed to rest outdoors. These exophilic populations likely represented what would now be recognized as the Kiribina form of *An. funestus*.

More recently, further epidemiologically significant behavioural heterogeneities in *An. funestus* from the same biogeographical area have been recognized following large-scale implementation of indoor-based vector control interventions. After mass deployment of insecticide-treated bed nets, the biting cycle of *An. funestus* shifted from its usual peak between 02:00 and 04:00 toward a later peak between dawn and early morning hours, when human hosts are less likely to be protected by nets [38]. Unfortunately, it is not known whether this behavioural shift was associated with a change in the chromosomal composition of the local *An. funestus* population.

The Folonzo and Kiribina chromosomal forms have been well characterized across several hundred kilometres and all ecozones of Burkina Faso [16,23]. However, their broader geographical distribution in Africa is poorly known. Certainly, they occur as far west as Senegal [15,27,39]. A recent study of sympatric populations of these forms, the first of its kind in Senegal, found stable co-existence of the forms across three successive breeding seasons and concluded, in accord with the present study, that Kiribina predominated, and rates of anthropophagy and sporozoite infection were comparable between forms, although both metrics were considerably lower in Senegal (~30 and ~3%, respectively) than they were in Burkina Faso [27]. Unfortunately, due to very low outdoor resting sample size (five total, of which only three could be identified chromosomally as Kiribina), indoor/outdoor resting behaviour was difficult to compare between forms, and thus, between studies. Cameroon is the most easterly country in which *An. funestus* chromosomal forms have been reported [36], but their vectorial heterogeneities (if any) are essentially uncharacterized. Available cytogenetic data suggest that these forms are largely allopatric in Cameroon, with Folonzo occurring in the mesic, forested south and Kiribina to the north in the dry savannas, except for a central contact zone at the forest-savanna transition, where stable sympatric co-existence of the two forms has not been clearly resolved [35-37]. In another parallel with the *An. gambiae* chromosomal forms, there is no evidence for the co-occurrence of *An. funestus* chromosomal forms in East Africa [40]; existing populations of *An. funestus* in eastern Africa are hypothesized to be allied with the Folonzo form [16], although that proposal has yet to be tested genetically.

Ample indication now exists of the practical importance of population structure and behavioural heterogeneities hidden within *An. funestus,* for malaria epidemiology and control in West Africa, if not beyond. In this light, the dearth of information about the wider geographic distribution and associated bionomics and vectorial parameters of the Folonzo and Kiribina forms is a problem that must be remedied as a matter of priority. The polytene chromosomes of *An. funestus* are considerably more difficult to spread and analyse than those of *An. gambiae*, a factor that has impeded past research on *An. funestus.* The demanding and specialized task of polytene chromosome-based identification, the restrictive sex and life stage from which favourable chromosomes are obtained, and the absence of any known DNA-based diagnostics to distinguish the chromosomal forms, all but prohibit deeper field investigation of Folonzo and Kiribina, particularly studies of their larval biology which is presumed to be a driver of their ecological and behavioural divergence. Genome sequencing of *An. gambiae* in 2002 [41], and the discovery of molecular forms of *An. gambiae* detectable by a simple PCR assay [26], greatly transformed understanding of the complexities of *An. gambiae* population structure and its impacts on malaria transmission. Recent whole genome sequencing and a newly available reference assembly for *An. funestus*[42] offer a platform that will support a more detailed understanding of *An. funestus* population structure across Africa, as well as an efficient means to discover genomic sequences potentially useful for molecular taxonomy of Folonzo and Kiribina.

For decades, patterns of chromosomal inversion polymorphism have guided discovery of population structure and even species boundaries hidden inside otherwise morphologically indistinguishable groups of anopheline mosquitoes i.e., [16,43-46]. Such an association of inversions with population substructure could be an incidental consequence of genetic drift owing to reduced gene flow, or the result of demographic history, but the observation that polymorphic inversions are often clinally distributed with respect to environmental gradients and subject to repeating seasonal fluctuations in frequency suggests that they are subject to strong selective forces [47]. In anopheline mosquitoes, as in many animal and plant species, chromosomal inversions are implicated in local adaptation to environmental heterogeneities [35,48-51]. To the extent that speciation may occur as a by-product of adaptive divergence, chromosomal inversions may also be instrumental in lineage splitting, as proposed by Coluzzi for anopheline mosquitoes [52]. That Kiribina and Folonzo forms are characterized by alternative arrangements of chromosomal inversions, and that these alternative arrangements shift in relative frequency according to geography, season, and larval habitat availability, suggests a direct role for chromosomal rearrangements in adaptation to heterogeneous and changing environments (see also [35,50]). Thus, beyond simply serving as markers for epidemiologically relevant population structure, alternative chromosomal arrangements some how condition different physiological and behavioural responses to the environment. A mechanistic understanding of what the adaptations are and how they evolved could prove instrumental in predicting how *An. funestus* may be capable of responding to future environmental challenges, including anthropogenic changes to climate and landscape, and exposure to new means of vector control.

## Competing interests

The authors declare that they have no competing interests.

## Authors’ contributions

CC, NFS and NB conceived the study. WG performed the field collections. NFS provided logistical support throughout the study. WG performed the karyotyping. WG, CC and FL analysed results. WG, CC and NB wrote the manuscript. All authors read and approved the final manuscript.

## References

[B1] Sinka ME Bangs MJ Manguin S Rubio-Palis Y Chareonviriyaphap T Coetzee M Mbogo CM Hemingway J Patil AP Temperley WH Gething PW Kabaria CW Burkot TR Harbach RE Hay SI A global map of dominant malaria vectors Parasit Vectors 2012 5 69 10.1186/1756-3305-5-69 22475528 PMC3349467

[B2] Gillies MT De Meillon B The Anophelinae of Africa South of the Sahara 1968 2 Johannesburg: South African Institute for Medical Research

[B3] Coluzzi M Sabatini A Petrarca V Di Deco MA Chromosomal differentiation and adaptation to human environments in the *Anopheles gambiae* complex Trans R Soc Trop Med Hyg 1979 73 483 497 10.1016/0035-9203(79)90036-1 394408

[B4] White BJ Collins FH Besansky NJ Evolution of *Anopheles gambiae* in relation to humans and malaria Annu Rev Ecol Evol Syst 2011 42 111 132 10.1146/annurev-ecolsys-102710-145028

[B5] Garros C Harbach RE Manguin S Systematics and biogeographical implications of the phylogenetic relationships between members of the Funestus and Minimus groups of *Anopheles* (Diptera: Culicidae) J Med Entomol 2005 42 7 18 10.1603/0022-2585(2005)042[0007:SABIOT]2.0.CO;2 15691003

[B6] Harbach RE The classification of genus *Anopheles* (Diptera: Culicidae): a working hypothesis of phylogenetic relationships Bull Entomol Res 2004 94 537 553 15541193 10.1079/ber2004321

[B7] Coetzee M Koekemoer LL Molecular systematics and insecticide resistance in the major African malaria vector *Anopheles funestus* Annu Rev Entomol 2013 58 393 412 10.1146/annurev-ento-120811-153628 23317045

[B8] Spillings BL Brooke BD Koekemoer LL Chiphwanya J Coetzee M Hunt RH A new species concealed by *Anopheles funestus* Giles, a major malaria vector in Africa Am J Trop Med Hyg 2009 81 510 515 19706923

[B9] Hackett BJ Gimnig J Guelbeogo W Costantini C Koekemoer LL Coetzee M Collins FH Besansky NJ Ribosomal DNA internal transcribed spacer (ITS2) sequences differentiate *Anopheles funestus* and *An. rivulorum*, and uncover a cryptic taxon Insect Mol Biol 2000 9 369 374 10.1046/j.1365-2583.2000.00198.x 10971714

[B10] Cohuet A Simard F Toto J-C Kengne P Coetzee M Fontenille D Species identification within the *Anopheles funestus* group of malaria vectors in Cameroon and evidence for a new species Am J Trop Med Hyg 2003 69 200 205 13677376

[B11] Koekemoer LL Misiani EA Hunt RH Kent RJ Norris DE Coetzee M Cryptic species within *Anopheles longipalpis* from southern Africa and phylogenetic comparison with members of the *An. funestus* group Bull Entomol Res 2009 99 41 49 10.1017/S0007485308006123 18715522 PMC4118299

[B12] Sinka ME Bangs MJ Manguin S Coetzee M Mbogo CM Hemingway J Patil AP Temperley WH Gething PW Kabaria CW Okara RM Van Boeckel T Godfray HC Harbach RE Hay SI The dominant *Anopheles* vectors of human malaria in Africa, Europe and the Middle East: occurrence data, distribution maps and bionomic precis Parasit Vectors 2010 3 117 10.1186/1756-3305-3-117 21129198 PMC3016360

[B13] Sharakhov I Braginets O Grushko O Cohuet A Guelbeogo WM Boccolini D Weill M Costantini C Sagnon N Fontenille D Yan G Besansky NJ A microsatellite map of the African human malaria vector *Anopheles funestus* J Hered 2004 95 29 34 10.1093/jhered/esh011 14757727

[B14] Green CA Hunt RH Interpretation of variation in ovarian polytene chromosomes of Anopheles funestus Giles, A. parensis Gillies, and A. aruni? Genetica 1980 51 187 195 10.1007/BF00121610

[B15] Lochouarn L Dia I Boccolini D Coluzzi M Fontenille D Bionomical and cytogenetic heterogeneities of *Anopheles funestus* in Senegal Trans R Soc Trop Med Hyg 1998 92 607 612 10.1016/S0035-9203(98)90782-9 10326101

[B16] Costantini C Sagnon NF Ilboudo-Sanogo E Coluzzi M Boccolini D Chromosomal and bionomic heterogeneities suggest incipient speciation in *Anopheles funestus* from Burkina Faso Parassitologia 1999 41 595 611 10870569

[B17] Guelbeogo WM Grushko O Boccolini D Ouedraogo PA Besansky NJ Sagnon NF Costantini C Chromosomal evidence of incipient speciation in the Afrotropical malaria mosquito *Anopheles funestus* Med Vet Entomol 2005 19 458 469 10.1111/j.1365-2915.2005.00595.x 16336311

[B18] Michel AP Ingrasci MJ Schemerhorn BJ Kern M Le Goff G Coetzee M Elissa N Fontenille D Vulule J Lehmann T Sagnon N Costantini C Besansky NJ Rangewide population genetic structure of the African malaria vector *Anopheles funestus* Mol Ecol 2005 14 4235 4248 10.1111/j.1365-294X.2005.02754.x 16313589

[B19] Koekemoer LL Kamau L Garros C Manguin S Hunt RH Coetzee M Impact of the Rift Valley on restriction fragment length polymorphism typing of the major African malaria vector *Anopheles funestus* (Diptera: Culicidae) J Med Entomol 2006 43 1178 1184 10.1603/0022-2585(2006)43[1178:IOTRVO]2.0.CO;2 17162950

[B20] Choi KS Koekemoer LL Coetzee M Population genetic structure of the major malaria vector Anopheles funestus s.s. and allied species in southern Africa Parasit Vectors 2012 5 283 10.1186/1756-3305-5-283 23216696 PMC3533957

[B21] Coluzzi M Petrarca V DiDeco MA Chromosomal inversion intergradation and incipient speciation in *Anopheles gambiae* Boll Zool 1985 52 45 63 10.1080/11250008509440343

[B22] Guelbeogo WM Sagnon N Grushko O Yameogo MA Boccolini D Besansky NJ Costantini C Seasonal distribution of *Anopheles funestus* chromosomal forms from Burkina Faso Malar J 2009 8 239 10.1186/1475-2875-8-239 19857258 PMC2775746

[B23] Michel AP Grushko O Guelbeogo WM Lobo NF Sagnon N Costantini C Besansky NJ Divergence with gene flow in *Anopheles funestus* from the Sudan Savanna of Burkina Faso, West Africa Genetics 2006 173 1389 1395 10.1534/genetics.106.059667 16648581 PMC1526678

[B24] Michel AP Guelbeogo WM Grushko O Schemerhorn BJ Kern M Willard MB Sagnon N Costantini C Besansky NJ Molecular differentiation between chromosomally defined incipient species of *Anopheles funestus* Insect Mol Biol 2005 14 375 387 10.1111/j.1365-2583.2005.00568.x 16033431

[B25] Coetzee M Hunt RH Wilkerson R Della Torre A Coulibaly MB Besansky NJ *Anopheles coluzzii* and *Anopheles amharicus*, new members of the *Anopheles gambiae* complex Zootaxa 2013 3619 246 274 26131476

[B26] della Torre A Fanello C Akogbeto M Dossou-yovo J Favia G Petrarca V Coluzzi M Molecular evidence of incipient speciation within *Anopheles gambiae s.s.* in West Africa Insect Mol Biol 2001 10 9 18 10.1046/j.1365-2583.2001.00235.x 11240632

[B27] Dia I Sagnon N Guelbeogo MW Diallo M Bionomics of sympatric chromosomal forms of *Anopheles funestus* (Diptera: Culicidae) J Vector Ecol 2011 36 343 347 10.1111/j.1948-7134.2011.00174.x 22129405

[B28] Gillies MT Coetzee M A Supplement to the Anophelinae of Africa South of the Sahara 1987 Johannesburg: The South African Institute for Medical Research

[B29] Della Torre A Crampton JM, Beard CB, Louis C Polytene chromosome preparation from anopheline mosquitoes Molecular Biology of Disease Vectors: A Methods Manual 1997 London: Chapman & Hall 329 336

[B30] Service MW Mosquito Ecology. Field Sampling Methods 1993 2 London: Elsevier Applied Science Publishers, Ltd

[B31] Bland JM Altman DG Statistics notes. The odds ratio Br Med J 2000 320 1468 10.1136/bmj.320.7247.1468 10827061 PMC1127651

[B32] Powell JR Petrarca V della Torre A Caccone A Coluzzi M Population structure, speciation, and introgression in the *Anopheles gambiae* complex Parassitologia 1999 41 101 113 10697841

[B33] Molineaux L Grammicia G The Garki Project. Research on the Epidemiology and Control of Malaria in the Sudan Savanna of West Africa 1980 Geneva, Switzerland: World Health Organization

[B34] Zahar AR Vector Bionomics in the Epidemiology and Control of Malaria 1985 Geneva, Switzerland: World Health Organization

[B35] Ayala D Fontaine MC Cohuet A Fontenille D Vitalis R Simard F Chromosomal inversions, natural selection and adaptation in the malaria vector *Anopheles funestus* Mol Biol Evol 2011 28 745 758 10.1093/molbev/msq248 20837604 PMC3002248

[B36] Cohuet A Dia I Simard F Raymond M Rousset F Antonio-Nkondjio C Awono-Ambene PH Wondji CS Fontenille D Gene flow between chromosomal forms of the malaria vector *Anopheles funestus* in Cameroon, Central Africa, and its relevance in malaria fighting Genetics 2005 169 301 311 15677749 10.1534/genetics.103.025031 PMC1448888

[B37] Dia I Boccolini D Antonio-Nkondjio C Costantini C Fontenille D Chromosomal inversion polymorphism of *Anopheles funestus* from forest villages of South Cameroon Parassitologia 2000 42 227 229 11686083

[B38] Moiroux N Gomez MB Pennetier C Elanga E Djenontin A Chandre F Djegbe I Guis H Corbel V Changes in *Anopheles funestus* biting behavior following universal coverage of long-lasting insecticidal nets in Benin J Inf Dis 2012 206 1622 1629 10.1093/infdis/jis565 22966127

[B39] Dia I Lochouarn L Boccolini D Costantini C Fontenille D Spatial and temporal variations of the chromosomal inversion polymorphism of *Anopheles funestus* in Senegal Parasite 2000 7 179 184 11031753 10.1051/parasite/2000073179

[B40] Kamau L Hunt R Coetzee M Analysis of the population structure of *Anopheles funestus* (Diptera: Culicidae) from western and coastal Kenya using paracentric chromosomal inversion frequencies J Med Entomol 2002 39 78 83 10.1603/0022-2585-39.1.78 11931275

[B41] Holt RA Subramanian GM Halpern A Sutton GG Charlab R Nusskern DR Wincker P Clark AG Ribeiro JM Wides R Salzberg SL Loftus B Yandell M Majoros WH Rusch DB Lai Z Kraft CL Abril JF Anthouard V Arensburger P Atkinson PW Baden H de Berardinis V Baldwin D Benes V Biedler J Blass C Bolanos R Boscus D Barnstead M The genome sequence of the malaria mosquito *Anopheles gambiae* Science 2002 298 129 149 10.1126/science.1076181 12364791

[B42] Neafsey DE Christophides GK Collins FH Emrich SJ Fontaine MC Gelbart W Hahn MW Howell PI Kafatos FC Lawson D Muskavitch MA Waterhouse RM Williams LJ Besansky NJ The evolution of the Anopheles 16 genomes project G3 2013 3 1191 1194 2013 23708298 10.1534/g3.113.006247 PMC3704246

[B43] Subbarao SK Anopheles Species Complexes in South and South-East Asia. SEARO Technical Publication No. 57 2007 New Delhi: World Health Organization, Regional Office for South-East Asia

[B44] Toure YT Petrarca V Traore SF Coulibaly A Maiga HM Sankare O Sow M DiDeco MA Coluzzi M The distribution and inversion polymorphism of chromosomally recognized taxa of the *Anopheles gambiae* complex in Mali, West Africa Parassitologia 1998 40 477 511 10645562

[B45] Coluzzi M Sabatini A Della Torre A Di Deco MA Petrarca V A polytene chromosome analysis of the *Anopheles gambiae* species complex Science 2002 298 1415 1418 10.1126/science.1077769 12364623

[B46] Kitzmiller JB Frizzi G Baker RH Wright JW, Pal R Evolution and speciation within the *Maculipennis* Complex of the genus *Anopheles* Genetics of Insect Vectors of Disease 1967 Amsterdam: Elsevier

[B47] Hoffmann AA Rieseberg LH Revisiting the impact of inversions in evolution: from population genetic markers to drivers of adaptive shifts and speciation? Ann Rev Ecol Evol Syst 2008 39 21 42 10.1146/annurev.ecolsys.39.110707.173532 20419035 PMC2858385

[B48] Kirkpatrick M Barton N Chromosome inversions, local adaptation and speciation Genetics 2006 173 419 434 10.1534/genetics.105.047985 16204214 PMC1461441

[B49] Kirkpatrick M How and why chromosome inversions evolve PLoS Biol 2010 8 9 e1000501 doi:10.1371/journal.pbio.1000501 10.1371/journal.pbio.1000501 20927412 PMC2946949

[B50] Ayala D Guerrero RF Kirkpatrick M Reproductive isolation and local adaptation quantified for a chromosome inversion in a malaria mosquito Evolution 2013 67 946 958 10.1111/j.1558-5646.2012.01836.x 23550747

[B51] Cheng C White BJ Kamdem C Mockaitis K Costantini C Hahn MW Besansky NJ Ecological genomics of *Anopheles gambiae* along a latitudinal cline: a population-resequencing approach Genetics 2012 190 1417 1432 10.1534/genetics.111.137794 22209907 PMC3316653

[B52] Coluzzi M Barigozzi C Spatial distribution of chromosomal inversions and speciation in anopheline mosquitoes Mechanisms of Speciation 1982 New York: Alan R. Liss, Inc 143 153 7178155

